# Moving beyond the mousetrap: current and emerging humanized mouse and rat models for investigating prevention and cure strategies against HIV infection and associated pathologies

**DOI:** 10.1186/s12977-020-00515-3

**Published:** 2020-04-10

**Authors:** Yash Agarwal, Cole Beatty, Shivkumar Biradar, Isabella Castronova, Sara Ho, Kevin Melody, Moses Turkle Bility

**Affiliations:** 1 Department of Infectious Diseases and Microbiology, Graduate School of Public Health, University of Pittsburgh, Pittsburgh, PA USA; 2 Galveston National Laboratory and Department of Microbiology and Immunology, University of Texas Medical Branch, Galveston, TX USA

**Keywords:** Humanized mouse and rat models, Modeling HIV infection and reservoir, Modeling human anti-HIV immune response, In vivo models for HIV cure, Animal models for HIV vaccines

## Abstract

The development of safe and effective combination antiretroviral therapies for human immunodeficiency virus (HIV) infection over the past several decades has significantly reduced HIV-associated morbidity and mortality. Additionally, antiretroviral drugs have provided an effective means of protection against HIV transmission. Despite these advances, significant limitations exist; namely, the inability to eliminate HIV reservoirs, the inability to reverse lymphoid tissues damage, and the lack of an effective vaccine for preventing HIV transmission. Evaluation of the safety and efficacy of therapeutics and vaccines for eliminating HIV reservoirs and preventing HIV transmission requires robust in vivo models. Since HIV is a human-specific pathogen, that targets hematopoietic lineage cells and lymphoid tissues, in vivo animal models for HIV-host interactions require incorporation of human hematopoietic lineage cells and lymphoid tissues. In this review, we will discuss the construction of mouse models with human lymphoid tissues and/or hematopoietic lineage cells, termed, human immune system (HIS)-humanized mice. These HIS-humanized mouse models can support the development of functional human innate and adaptive immune cells, along with primary (thymus) and secondary (spleen) lymphoid tissues. We will discuss applications of HIS-humanized mouse models in evaluating the safety and efficacy of therapeutics against HIV reservoirs and associated immunopathology, and delineate the human immune response elicited by candidate HIV vaccines. In addition to focusing on how these HIS-humanized mouse models have already furthered our understanding of HIV and contributed to HIV therapeutics development, we discuss how emerging HIS-humanized rat models could address the limitations of HIS-mouse models.

## Background

**Fig. 1:**
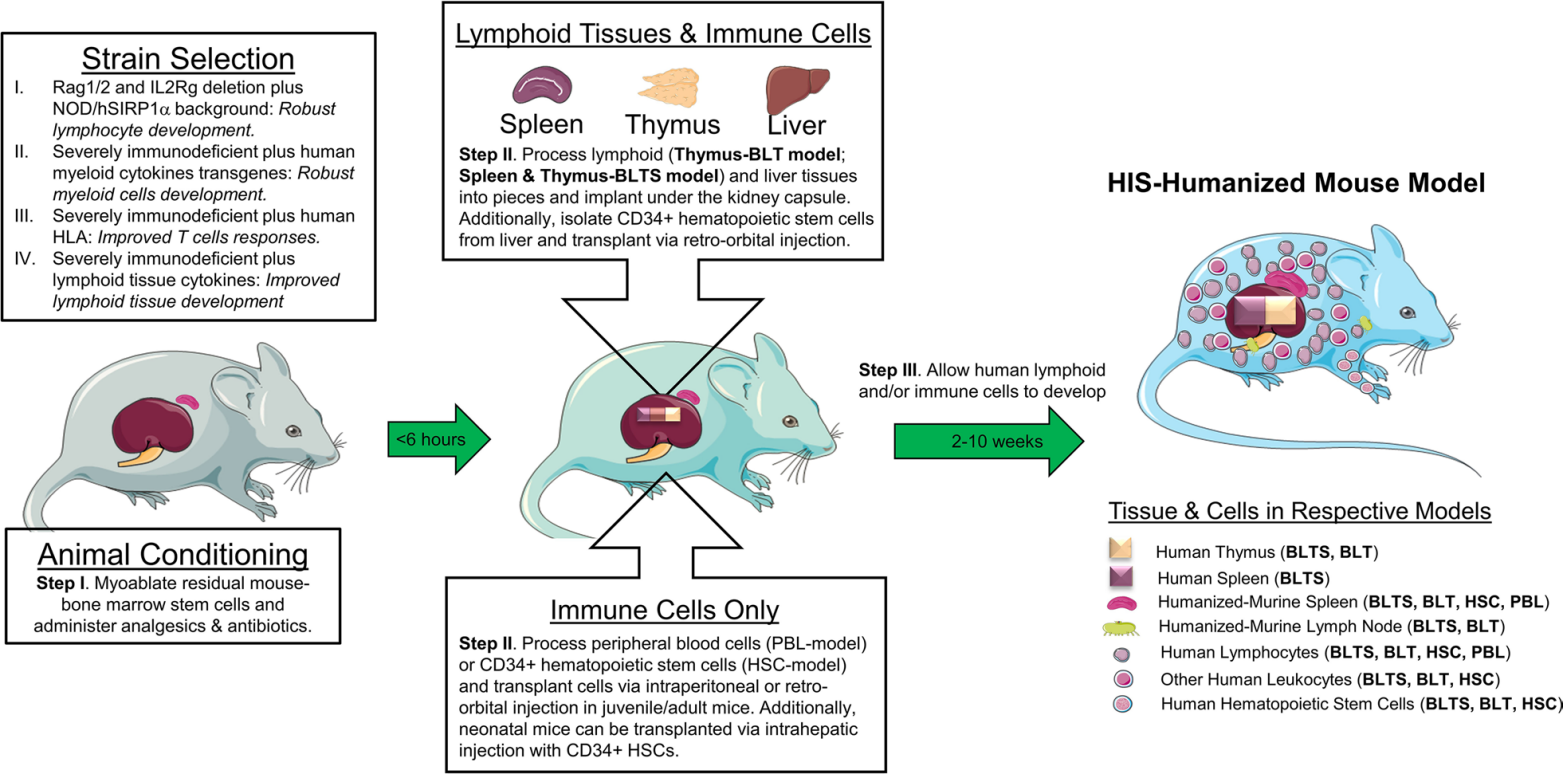
Construction of the human Immune System-humanized mouse models. (I) Immunodeficient mice are myoablated via irradiation or busulfan, followed by the administration of antibiotics and analgesics. General anesthesia is induced prior to surgery. (II) To generate human lymphoid tissue xenografts along with autologous immune cell reconstitution, human fetal lymphoid tissue(s) and liver are processed into 1 mm^2^ pieces, and autologous CD34 + HSCs are isolated from the fetal liver via immunomagnetic selection. CD34 + HSCs are then transplanted via retro-orbital injection following renal capsule transplantation of the lymphoid tissues. Alternatively, to generate human immune cell only, PBMCs, CD4+ T cells, or CD34 + HSCs are transplanted via IV or IP injection. (III) Transplanted mice are maintained under specific pathogen-free conditions and the human lymphoid tissue(s), and/or immune cell reconstitution in the peripheral blood and murine lymphoid tissues (humanized-murine tissues) are allowed to develop over a period of 2–10 weeks (or more), resulting in the HIS-humanized mouse model

Despite combination antiretroviral therapy (cART)-mediated suppression of human immunodeficiency virus (HIV) replication and promotion of immune reconstitution in patients, HIV-associated morbidity persists and is associated with the latent reservoir, unresolved immune abnormalities, and fibrosis in lymphoid organs [1]. Additionally, HIV transmission remains endemic across the globe, and development of a functional cure and/or an effective vaccine will be required to end this epidemic [2]. HIV is a human specific pathogen; thus, animal models for evaluating the safety and efficacy of therapeutics and vaccines directly against HIV requires the incorporation of human lymphoid tissues and/or hematopoietic lineage cells. Such mouse models exist, and are termed, human immune system (HIS)-humanized mice [3, 4]. To construct HIS-humanized mice, immunodeficient mice are myoablated to eradicate residual mouse bone marrow stem cells, and then engrafted with human peripheral blood mononuclear cells (PBMCs) or human hematopoietic stem cells (HSC) with or without transplantation of lymphoid tissues, such as thymus and/or spleen [5]. Over a period of a few weeks to months, transplanted mice develop human immune cells, and reconstitution is confirmed by flow cytometry [5] (Fig. 1). HIS-humanized mice can then be employed in studies investigating HIV prevention or cure strategies [5]. In this review, we will discuss the myriad of approaches for developing HIS-humanized mouse models, and their applications in HIV therapeutics and vaccine development studies, along with their limitations. Finally, we will discuss the potential of an emerging HIS-humanized rat model, which has a longer lifespan and greater physiological similarity to humans compared to mice, designed to enable longitudinal studies in evaluating therapeutics against HIV reservoirs and vaccine-induced immunity [6, 7].

## Humanized mice that incorporate human immune cells

### Peripheral blood lymphocytes (PBL)-humanized mouse model

Human CD4^+^ T cells are the major target for HIV infection; thus, a mouse model with human CD4^+^ T cells provides a platform for modeling HIV/AIDS. Various immunodeficient mouse models lacking mature T, B, [8] and NK cells, along with defects in macrophage phagocytic function [9] support robust reconstitution of human CD4+ T cells and other lymphocytes (e.g. CD8 + T cells) following transplantation of human PBMCs or CD4+ T cells. Such cells can be transplanted via intravenous (IV) or intraperitoneal (IP) injection into myoablated, immunodeficient juvenile mice (6–8 weeks old) at a dose of 5–10 × 10^6 cell per mouse, to generate peripheral blood lymphocyte (PBL)-humanized mice. Human CD4 + T cells are readily detectable in the blood at 4 weeks post-transplantation [9, 10], hence providing a humanized mouse model that can be generated in a relatively short period. The PBL-humanized mouse model supports HIV replication and provides a means of evaluating the efficacy of direct-acting therapeutics (e.g. antivirals drugs, antibodies) geared towards preventing HIV transmission [11] and controlling HIV replication [9]. Additionally, PBL-humanized mouse models constructed using PBMCs from HIV-infected individuals with undetectable viral load can be employed as an in vivo assay (mouse-quantitative viral outgrowth assay) for evaluating the eradication of the HIV reservoir in said individuals [12, 13]. A major limitation of this model is the rapid development of graft-versus-host (GvHD) disease within 6–7 weeks following transplantation of lymphocytes, thus significantly restricting the experimental window [14]. Additionally, the PBL-humanized mouse model does not incorporate human macrophages, which are a major HIV reservoir in various organs, including the brain [9, 15]. However, modification of the PBL-humanized mouse model via transplantation of HIV-infected monocyte-derived macrophages in the brain supports HIV infection and pathogenesis in the brain [15].

### Hematopoietic stem cells (HSC)-humanized mouse model

In order to generate a de novo human immune system and reconstitute a broader spectrum of human immune cells in HIS-humanized mice, myoablated, immunodeficient mice are transplanted with human CD34 + HSCs via intrahepatic or intracardiac injection in neonatal mice [10, 16] or IV injection in juvenile/adult mice [16, 17]. These HSCs can be obtained from a myriad of sources, including fetal liver tissue [17, 18] and neonatal cord blood cells [4, 16, 19]. Human immune reconstitution in the HSC-humanized mouse model requires 10–12 weeks to develop [20]. Various hematopoietic lineages, including T cells, monocytes/macrophages, B cells and dendritic cells are developed in the blood and other tissues (e.g., spleen, liver, brain) [20]. Moreover, the HSC-humanized mouse model generates a naïve human immune system, which negates confounding factors associated with prior pathogen exposure [16, 21]. The HSC-humanized mouse model supports HIV infection, CD4+ T cell depletion, chronic immune activation and limited anti-HIV T and B cell immune responses [4]. A major advantage of the HSC-humanized mouse model over the PBL-humanized mouse model is the delayed and reduced incidence of GvHD, which provides the opportunity for long-term modeling of HIV infection and replication [9]. The HSC-humanized mouse model provides a means of evaluating the efficacy and safety of direct-acting therapeutics (e.g. antivirals drugs, antibodies) and immune-modulatory agents (e.g. pDC modulators [22]) geared towards preventing HIV transmission, controlling HIV replication, and ameliorating CD4+ T cell depletion and chronic immune activation [4]. The HSC-humanized mouse model supports HIV transmission via the IV (along with IP) route [18]; however, conflicting reports exist for the mucosal route of transmission [23, 24]. Additionally, the reconstituted human T cells are educated in the mouse thymic epithelium, thus limiting antigen-specific responses [25]. This limitation of T cell education in the murine thymic epithelium has been partially overcome by the construction human leukocyte antigen (HLA) class I transgenic-immunodeficient mice to support robust T cell development of HLA-matched HSC transplants [26]. Moreover, lymph nodes and spleen are poorly reconstituted, including a limited development of human B and myeloid cells in the white and red pulps of the spleen [27]. Several modifications have been made to the HSC model to address these limitations. Li et al. constructed an immunodeficient mouse model that incorporated a lymphoid tissue-stromal cytokine transgene (i.e. thymic-stromal-cell-derived lymphopoietin) and demonstrated improved lymph node development in HSC-humanized mice [27]. Additionally, studies have demonstrated enhanced human B and myeloid cell development in murine secondary lymphoid tissues via transgenic expression of critical cytokines (i.e. IL6; IL3, GM-CSF and SCF) for B and myeloid cell maturation [28–30]. Although incorporation of requisite human transgenes in HIS-humanized mice has been successful in demonstrating improved development of immune cells, often the resultant lineage is skewed, as the transgene expression is not synchronized for physiological expression and supporting stromal cells and other essential cytokines are absent [28–30].

## Humanized mice that incorporate human immune cells and lymphoid tissues

### Bone marrow–liver–thymus (BLT)-humanized mouse model

**Fig. 2:**
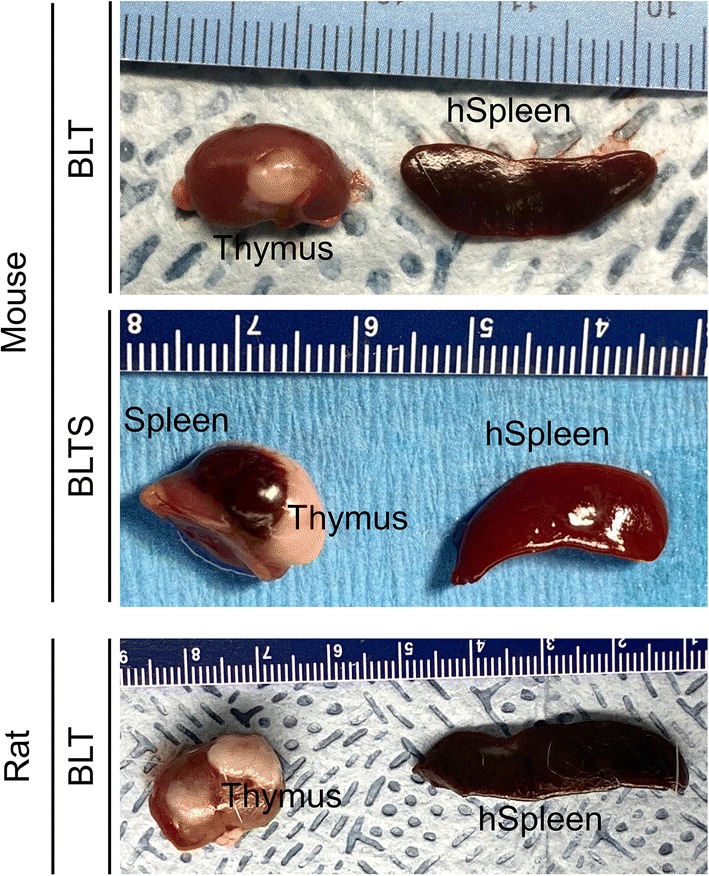
Current and emerging HIS-humanized animal models. To construct HIS-humanized mice and rats, immunodeficient mice and rats are myoablated, followed by engraftment of human lymphoid tissues (thymus with or without human spleen) under the kidney capsule, along with injection of autologous human CD34+ hematopoietic stem cells. In representative images, we show the engrafted human lymphoid tissues (human thymus xenograft-thymus, white tissue; human spleen xenograft-Spleen, dark brown tissue and the reconstituted rodent-spleen (humanized spleen-hSpleen). Note: mouse and rat organs are not at the same scale

Another strategy for improving human immune cell development in HIS-humanized mouse models is to implant human lymphoid tissues containing the requisite microenvironment for supporting robust immune cell development. To facilitate human T cell education and associated function, human thymic tissues are incorporated in HIS-humanized mice, and termed, Bone marrow–liver–thymus (BLT)-humanized mice [31, 32] (Fig. 2). BLT-humanized mice have served as a key animal model for HIV research for over a decade and are a cost-effective alternative to the surrogate, simian immunodeficiency virus (SIV)-non-human primate (NHP) models. The BLT-humanized mouse model is generated by surgically transplanting myoablated, immunodeficient mice with fetal human liver and thymus tissues, followed by IV injection of autologous CD34^+^ HSCs [31, 33, 34]. Transplanted mice require 10–12 weeks for systemic reconstitution of human cells post-transplantation [31, 33, 34]. The most widely utilized strain for constructing BLT mice is the NOD-*Prkdc*^*scid*^*IL2rg*^*Tm1Wjl*^ (NSG) [21, 32], which is readily available from Jackson Laboratory. BLT-humanized mice can also be constructed using comparable immunodeficient mouse strains, such as, C57BL/6 Rag2−/−γc−/−CD47−/− (TKO) [21, 35]. The key benefit of the BLT-humanized mouse model over PBL- and HSC-humanized mouse models is the presence of human thymic microenvironment, which facilitates T cell education in an autologous human tissue that contains the requisite stromal cells (as well as cytokines and factors, presumably at physiological levels) [21]. BLT-humanized mice have systemic tissue reconstitution with human immune cells, including in mucosal tissues, which enables mucosal transmission [36–43] and recapitulates the main route of HIV transmission in humans [36–44]. Other hallmarks of HIV infection and replication in BLT-humanized mice include robust T cell depletion [36, 42], central nervous system infiltration [45, 46], immune response [35, 47–50], and latency [51–53]. The BLT-humanized mouse model is a robust platform for evaluating HIV prevention and cure strategies, including antiretroviral therapy, pre-exposure prophylaxis (PrEP), latency reversing agents (LRA), vaccination, proviral excision, and T cell engineering (Table 1).

**Table 1:** Studies utilizing BLT-humanized mice for evaluating HIV-therapeutics

Strategy	Therapeutic agent(s)	Reference(s)
Antiviral therapy	dCA	[45, 56]
	EFdA	[57]
	RAL	[45]
	PD-1 mAb	[58]
	PG16 bNAb	[59]
	PGT121 bNAb	[60]
	3TC, TDF	[61]
	AZT, ddI, IDV	[52]
	FTC, RPV, DTG	[46, 57]
	FTC, TAF, EVG	[62]
	FTC, TDF, DTG	[37, 46, 51, 53, 63]
	FTC, TDF, RAL	[37, 46, 51, 63]
	FTC, TDF, RAL, 3B3(Fv)-PE38 immunotoxin	[64]
	FTC, TDF, RAL, IFNα14	[61, 65]
Pre-exposure prophylaxis (PrEP)	C5A peptide	[66]
	Cc-griffithsin	[67]
	CD4 AsiCs	[68]
	CD4-expressing *Lactobacillus acidophilus*	[69]
	CD4mc P-III-48	[70]
	DTG-ultra LA	[71]
	EFdA	[72]
	G2-S16 PCD	[73]
	IgA	[74]
	MVC	[75]
	RAL-LA	[76]
	RPV-LA	[39, 77]
	siCCR5 LFA-1 I-tsNP	[78]
	TNV gel	[79–81]
	VRC01 bNAb	[82]
	FTC, TAF	[83]
	FTC, TDF	[36, 43, 84, 85]
	TAF, EVG	[86]
	b12, VRC01, VRC07 G54W bNAbs	[87]
Latency-reversing agents (LRAs)	AZD5582	[88]
	panobinostat	[89]
	SUW133 (bryostatin analog)	[90]
Vaccines	PLGA-Gag microparticles	[49, 91]
	Recombinant GP140∆683	[49, 91]
Proviral excision	saCas9/sgRNA	[92]
T cell engineering	CCR5 shRNA	[93, 94]
	CD4 CAR	[95]
3TC, lamivudine; AsiCs, aptamer-siRNA chimeras; AZT, zidovudine; bNAb, broadly neutralizing antibody; CCR5, C–C chemokine receptor type 5; CAR, chimeric antigen receptor; CD4, cluster of differentiation 4; CD4mc, CD4 mimetic compound; Cc, *Caulobacter crescentus* recombinant expressing; dCA, didehydro-Cortistatin A; ddI, didanosine; DTG, dolutegravir; DTG-ultra LA, long acting dolutegravir; EFdA, 4′-ethynyl-2-fluoro-2′-deoxyadeno-sine; EVG, elvitegravir; FTC, emtricitabine; IDV, indinavir; IFNα14, interferon α suptype 14; IgA, immunoglobulin A; LFA-1 I-tsNP, lymphocyte function–associated antigen-1 integrin-targeted and stabilized nanoparticle; mAb, monoclonal antibody; MVC, maraviroc; PCD, polyanionic carbosilane dendrimers; PD-1, programmed cell death protein 1; PLGA, poly(lactic-co-glycolic) acid; RAL, raltegravir; RAL-LA, long-acting raltegravir; RPV, rilpivirine; RPV-LA, long acting rilpivirine; saCas9/sgRNA, Staphylococcus aureus CRISPR-associated protein 9/single-guide RNA; shRNA, short hairpin RNA; siCCR5, small interfering RNA CCR5; TAF, tenofovir alafenamide; TDF, tenofovir disoproxil fumarate; TNV; tenofovir		

Despite significant advances gained from the BLT-humanized mouse model, the system does have some disadvantages. Construction of BLT-humanized mice requires advanced surgical expertise and extensive experience; therefore, these animals are constructed predominantly by specialized core facilities. Additionally, BLT-humanized mice are prone to GvHD, which limits the experimental window these animals can be utilized to approximately 6 months post-engraftment [54, 55]. However, BLT-humanized mice constructed with a C57BL/6 immunodeficient background are resistant to GvHD [35, 53]. Another disadvantage involves the use of human fetal tissues in constructing the model; these tissues are not readily available. Furthermore, a typical human fetal thymus and autologous fetal liver-derived HSCs can only support the construction of 15–25 BLT-humanized mice. The limited availability of said human fetal tissues creates logistical and operational constraints. Recently, a novel BLT-like humanized mouse model has been developed using non-autologous human cord blood-derived HSCs and human neonatal/pediatric thymus, which enable investigators to construct > 1000 BLT-like humanized mice using cryopreserved thymus tissues and readily available cord blood-derived HSCs [96]. Recent studies demonstrate that these BLT-like humanized mice develop human immune cells, support HIV infection and replication, and exhibit anti-HIV immune response (*unpublished data from Elie Haddad, Chloé Colas,* et al*., at the CanCure 5th Annual General Meeting—2019, Poster Session, in Montreal, Canada*). Despite systemic immune cell reconstitution and HIV-specific immune responses, both neonatal/pediatric tissue- and fetal tissue-derived BLT-humanized mouse models BLT mice do not develop a complete human immune system. The current widely used immunodeficient mouse models possess an IL-2 receptor γ chain deletion [97–100]. As a result, mouse lymphoid organs do not fully develop in such models, [21] and the loss of lymphoid tissue microenvironment impairs the ability of BLT-humanized mice to develop a robust humoral immune response, as immunoglobulins are skewed towards IgM or weak IgG response [35, 49, 99, 101–103]. Constructing BLT-humanized mice using immunodeficient mouse models with requisite human transgenic factors/cytokines, may optimize human B cell development and overcome the limitations of humoral immune response in the model [28, 104, 105]. An alternative strategy, which is consistent with the BLT-model strategy, is to incorporate the requisite human secondary lymphoid tissue (i.e. spleen) microenvironment for robust human immune cell (e.g. B cells, macrophages) development and response [5].

### Bone marrow–liver–thymus–spleen (BLTS)-humanized mouse model

To address the limitations of the BLT-humanized mouse model, namely, poor development of secondary lymphoid tissue and modest macrophage reconstitution, we incorporated human spleen into the BLT-humanized mouse model, and termed these animals, Bone marrow–liver–thymus–spleen (BLTS)-humanized mice [5] (Fig. 2). BLTS-humanized mice exhibit significant improvement over the BLT-humanized mice by addressing several limitations [5]. Successful spleen growth dramatically lowers the incidence of GvHD in BLTS-humanized mice, thus allowing for experimental studies that extend up to 9 months post-transplantation [5]. We speculate that the decreased GvHD in BLTS-humanized mice results from appropriate modulation of T cell activation by the human antigen-presenting cells in the human spleen xenograft. The human spleen in the BLTS-humanized mouse model recapitulates human adult spleen architecture and facilitates better reconstitution of immune cells, including human red pulp macrophages, which are poorly reconstituted in the BLT-humanized mouse model [5]. It is well established that macrophages can serve as a reservoir for HIV [106–108]; thus, the BLTS-humanized mouse model provides a system for investigating human splenic macrophage-HIV interactions [5]. Additionally, the spleen is a major lymphoid tissue reservoir, with B cell follicles in the white-pulp serving as an immune privilege site for anti-HIV T-cells [109]. The human spleen in BLTS-humanized mice provides a model for investigating anti-HIV immune response within the white-pulp and the role of B cell follicle in mediating HIV persistence. The BLTS-humanized mouse model supports cART-mediated HIV load suppression, and replication competent HIV reservoirs can be detected in human spleen tissues [5]. Lymphoid tissue fibrosis is an immuno-pathogenic feature associated with HIV infection and plays a major role in mediating chronic inflammation and abrogating the development of a robust immune response [110]. A major advantage of the BLTS-humanized mouse model is that HIV infection results in lymphoid tissue fibrosis; this disease manifestation is absent in HIV-infected BLT-humanized mice [5]. Although the BLTS-humanized mouse model exhibits more robust immune reconstitution compared to its BLT counterpart, the two models share some limitations. The transplantation of the human tissues under the renal capsule requires an individual with advanced surgical skills. The BLTS-humanized mouse model uses human fetal tissues, which introduces logistical and operational constraints. The use of frozen fetal tissues and HSCs can alleviate some of those constraints (*unpublished data*). Demonstrating robust anti-HIV immunity in HIS-humanized mouse models has been a long-term goal in the field because said system would allow robust evaluation of HIV vaccine candidates against circulating viral strains. The incorporation of human primary and secondary lymphoid tissues in HIS-humanized mice brings us closer to this goal. At present, we are actively investigating the anti-HIV human immunity in the BLTS-humanized mouse model to determine if this system provides a means for evaluating HIV vaccines.

## Emerging human immune system (HIS)-humanized rat model

Prior to the development of genetic engineering technologies for creating transgenic and knockout mice, rats were the predominant specie of rodents employed in biomedical research [6]. Advantages of using rats include their longer lifespans (~ 3.5 years) and larger size (~ 350 grams), which facilitates longer experimental window and larger sampling volumes compared to the short lifespan (< 1 year) and small size (< 25 g) of mice [6]. Rats provide a more ideal platform for in vivo imaging of diseases, as the larger size of rats provides better resolution [111]. Additionally, rat models exhibit advanced cognitive skills and critical physiological parameters (e.g. heart rate, drug metabolism) that more closely mimic humans [112–115]. Recent advances in genetic engineering, such as the CRISPR/Cas 9 technology has enabled the development of several immunodeficient rat models for transplanting and regenerating human tissues and cells [7, 116–118]. Similar to currently used immunodeficient mouse models, immunodeficient rat models carry mutations in Rag1/2 and IL2rγ genes, with or without SIRP1α transgene [7, 116–118]. A recent study demonstrated that these immunodeficient rats can be reconstituted with a myriad of human immune cells following transplantation with human-fetal liver-derived HSCs and autologous thymus tissues [7] (Fig. 2). These HIS-humanized rat models could provide a means for robust longitudinal studies on the safety and efficacy of therapeutic agents targeting the HIV reservoir. Additionally, HIS-humanized rats could provide a means for modeling HIV-associated end organ diseases, such as cardiovascular, neurocognitive and lung diseases.

## Conclusions

Despite successful prevention of HIV transmission with antiviral drugs, it is likely that an effective vaccine provides the only means of ending the HIV epidemic [2]. Over the past decades, several promising vaccine candidates have failed in large-scale safety and efficacy clinical trials [2]. The failure of those clinical trials suggests that improved “gate keeper” animal modeling systems are needed for better prediction of vaccine candidate outcomes in human clinical trials [119]. Currently, the surrogate SIV-NHP model is the sole “gate keeper” animal model for determining potential of vaccine candidates [119]. Candidate HIV vaccines selected using this gatekeeper system have been unsuccessful in human clinical trials [119]; suggesting, major improvements in animal modeling are needed. Although significant advancements are still needed in improving HIS-humanized models, several recent advances, such as improved human-secondary lymphoid tissue development, along with the previously developed, robust primary lymphoid tissue development has made it possible to evaluate human immune responses to vaccines [5, 27]. Ideally, these “improved” HIS-humanized mouse models will complement NHP models in addressing critical gaps, such as vaccine-induced immune responses against circulating HIV strains, vaccine safety in the context of HIV transmission, and *human*-correlates of immunity. Although cART has significantly reduced the morbidity and mortality associated with chronic HIV infection, the HIV reservoir persists in people living with HIV (PLHIV) and is associated with chronic inflammation, lymphoid tissue damage, and a myriad of end-organ diseases [1]. Therefore, eradicating the HIV reservoir and associated chronic inflammation and end organ diseases remains a major challenge. The mechanisms of HIV persistence in PLHIV, despite robust cART-mediated suppression of the virus, are thought to be multifactorial. These factors include persistence in transcriptionally quiescent resting memory CD4+ T cells in the peripheral blood and lymphoid tissues, infection of long-lived resident tissue macrophages in lymphoid tissues and immune privilege organs (e.g. brain, testes, B cell follicle, etc.), and dysregulation in anti-HIV immunity. By virtue of the multitude of factors that play a role in HIV persistence, in vivo models that recapitulate human host-HIV interactions are necessary for determining the safety and efficacy of therapeutic agents for eradicating HIV reservoirs. Improved HIS-humanized mouse models with systemic reconstitution of human immune cells and robust lymphoid tissues development provide a means of evaluating both direct-acting and immune-modulatory HIV-cure therapeutics. Further advances in improving the human-immune system in HIS-humanized mouse and rat models will provide better in vivo systems for evaluating the safety and efficacy of therapeutics and vaccines for HIV prevention and cure.

## Data Availability

Not applicable.

## Abbreviations

HIV: Human immunodeficiency virus
cART: Combination antiretroviral therapy
HIS: Human immune system
PBMCs: Peripheral blood mononuclear cells
PBL: Peripheral blood lymphocytes
HSC: Human hematopoietic stem cells (HSC)
GvHD: Graft-versus-host (GvHD)
IV: Intravenous
IP: Intraperitoneal
NSG: NOD-*Prkdc*^*scid*^*IL2rg*^*Tm1Wjl*^
TKO: C57BL/6 Rag2−/−γc−/−CD47−/−
BLT: Bone marrow–Liver-Thymus
BLTS: Bone marrow–liver–thymus–Spleen
